# Discovering variation of secondary metabolite diversity and its relationship with disease resistance in *Cornus florida* L.

**DOI:** 10.1002/ece3.4090

**Published:** 2018-05-04

**Authors:** Andrew L. Pais, Xu Li, Qiu‐Yun (Jenny) Xiang

**Affiliations:** ^1^ Department of Plant and Microbial Biology North Carolina State University Raleigh North Carolina; ^2^ Plants for Human Health Institute North Carolina State University Kannapolis North Carolina

**Keywords:** adaptation, *Cornus florida*, ecological genomics, population genetics, secondary metabolism, single nucleotide polymorphisms

## Abstract

Understanding intraspecific relationships between genetic and functional diversity is a major goal in the field of evolutionary biology and is important for conserving biodiversity. Linking intraspecific molecular patterns of plants to ecological pressures and trait variation remains difficult due to environment‐driven plasticity. Next‐generation sequencing, untargeted liquid chromatography–mass spectrometry (LC‐MS) profiling, and interdisciplinary approaches integrating population genomics, metabolomics, and community ecology permit novel strategies to tackle this problem. We analyzed six natural populations of the disease‐threatened *Cornus florida* L. from distinct ecological regions using genotype‐by‐sequencing markers and LC‐MS‐based untargeted metabolite profiling. We tested the hypothesis that higher genetic diversity in *C. florida* yielded higher chemical diversity and less disease susceptibility (screening hypothesis), and we also determined whether genetically similar subpopulations were similar in chemical composition. Most importantly, we identified metabolites that were associated with candidate loci or were predictive biomarkers of healthy or diseased plants after controlling for environment. Subpopulation clustering patterns based on genetic or chemical distances were largely congruent. While differences in genetic diversity were small among subpopulations, we did observe notable similarities in patterns between subpopulation averages of rarefied‐allelic and chemical richness. More specifically, we found that the most abundant compound of a correlated group of putative terpenoid glycosides and derivatives was correlated with tree health when considering chemodiversity. Random forest biomarker and genomewide association tests suggested that this putative iridoid glucoside and other closely associated chemical features were correlated to SNPs under selection.

## 1 INTRODUCTION

Plant secondary metabolites are closely tied to ecological functions and greatly affect community interactions (Dixon & Paiva, 1995; Moore, Andrew, Külheim, & Foley, 2014). Certain secondary metabolites may provide plants with specialized functions like deterrence to herbivory or infection (Harborne & Turner, 1984). Identifying the genetic basis of secondary compounds for such functions is of interest to the field of evolutionary ecology. Even small changes in genetic diversity may yield exceptionally large changes in secondary metabolism—producing novel molecules with often unknown biological activity (Firn & Jones, 2000). While specific compound classes such as iridoids, phenolics, and tannins are often the basis of study for ecological function in plants (Sardans, Peñuelas, & Rivas‐Ubach, 2011), secondary metabolite diversity as a trait, or chemotype, represents a special dimension of biodiversity important to natural and managed ecosystems (Bustos‐Segura et al., 2017). In contrast to the research presented in this article, few studies have evaluated broader relationships between chemical diversity and genetic diversity within species (focusing on the diversity of chemical compound composition among individuals in a population) while examining a select group of metabolites diagnostic of health versus disease (otherwise known as biomarkers) and associated with SNPs under selection. Recent innovations in next‐generation sequencing coupled with untargeted chemical profiling provide unique opportunities to examine these relationships in plant systems (Eckert et al., 2012; Gomez‐Casati, Zanor, & Busi, 2013; Raguso et al., 2015; Riedelsheimer et al., 2012). Integrating next‐generation sequencing technologies with population genomics and community ecology permits identification of chemical compounds and associated SNPs related to disease resistance or other ecologically functional traits.

Secondary metabolite richness and relative abundance of chemicals within individuals—a chemotype referred to as chemodiversity—are informative yet understudied metabolome properties helpful for understanding evolutionary and ecological processes (Hilker, 2014; Kellerman, Dittmar, Kothawala, & Tranvik, 2014). Promising work has investigated broader patterns of natural metabolome variation in the context of natural genetic variation, but most analyses of variation in chemical diversity focus on distance‐based measures versus explicit measurement of chemical richness. For example, significant correlations between metabolic and genetic distances were detected in nine *Arabidopsis thaliana* accessions exposed to different environments (Houshyani et al., 2012). In a second example, multigenerational lines inbred from different *Drosophila melanogaster* populations were found to remain distinguishable in general lipid composition, and approximately one‐fifth of the lipid compounds had clear concentration differences between male and female genotypes (Scheitz, Guo, Early, Harshman, & Clark, 2013). More recent studies of environmental and bud–leaf metabolome analyses of *Pinus pinaster* (ten European provenances in common garden) revealed two groups of individuals corresponding to spatially distinct regions (Meijón et al., 2016). All these studies used distance measures based on a reduced dimensionality of abundance differences for targeted compounds instead of explicitly calculating and comparing diversity indices (Appendix S1), which account for both chemical compounds’ presence–absence and relative abundances within each sample.

Studies employing diversity indices of broad chemical profiling are rare (Hilker, 2014), possibly due to previous aversion to adapting such indices outside of community ecology (Hurlbert, 1971). However, initial trepidations regarding usage of these indices are now being addressed with cautious interpretation of chemical diversity indices (Morris et al., 2014). Additional studies that integrate advancements in untargeted metabolomics (Alonso, Marsal, & Julià, 2015; Yi et al., 2016) and population‐landscape genomics (Anderson, Willis, & Mitchell‐Olds, 2011; Sork et al., 2013) with adoption of these chemical diversity indices would further demonstrate the power of this correlative approach to illustrate how genetics (i.e., locally adapted genes) and plant functional diversity (i.e., chemodiversity) influence plant health, after controlling for environment analytically.

**Figure 1:**
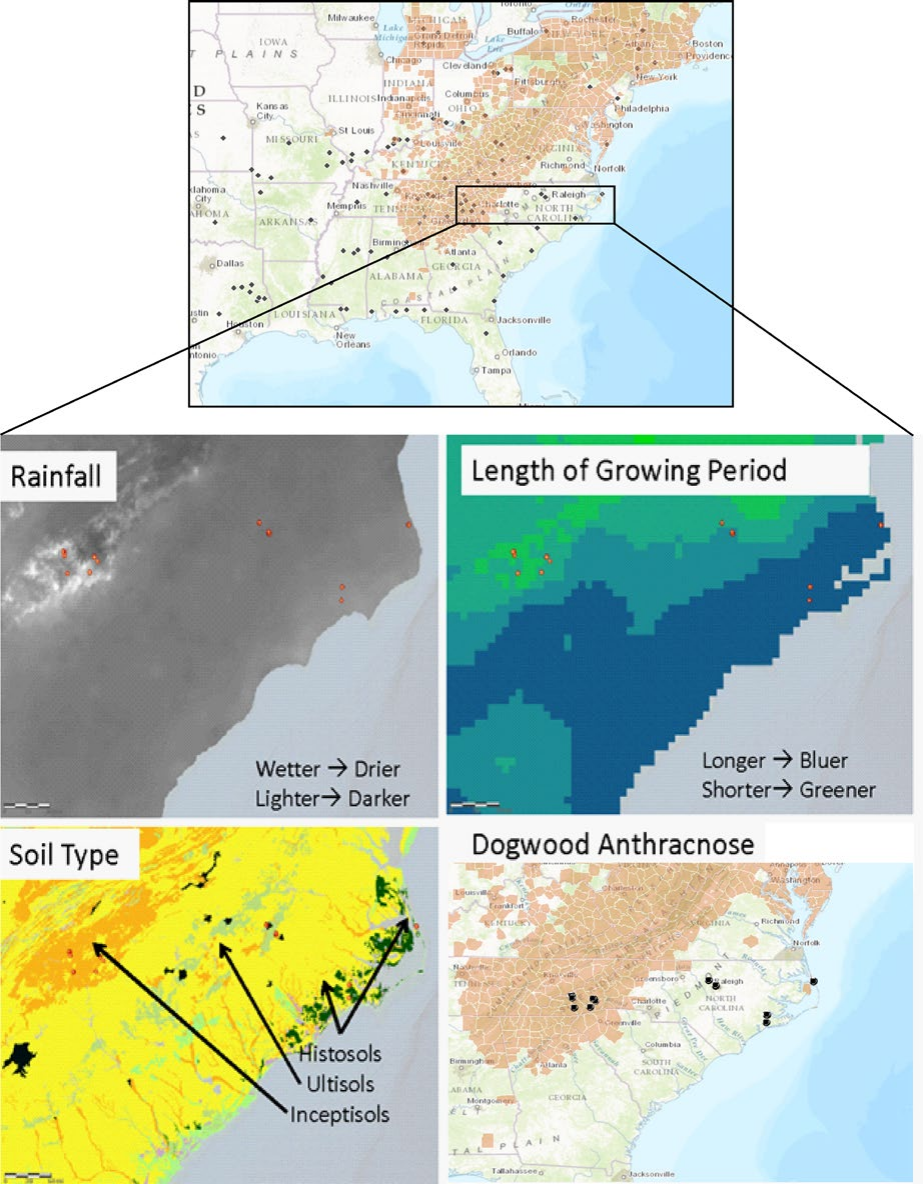
Subset of collections from broader study of *C. florida* (top) applied to metabolic study of chemical diversity in North Carolinian populations (bottom). Red counties have known incidence of dogwood anthracnose disease. For subset of populations sampled in this study, differences in mean monthly rainfall, length of growing period, soil type, and county occurrence of dogwood anthracnose are visualized to demonstrate the heterogeneity in environment that exists among the mountain, Piedmont, and Coastal Plain ecoregions of North Carolina

We use a multidisciplinary approach to characterize and evaluate how properties of genetic diversity and chemodiversity contribute to susceptibility or resistance to disease in *Cornus florida* (L.), the flowering dogwood tree. In addition, we have applied exploratory analyses to winnow an untargeted list of metabolites down to a select group of potential antimicrobial compounds—closely resembling compounds previously observed in dogwoods (He, Peng, Hamann, & West, 2014; Stermitz & Krull, 1998; Yue et al., 2006). The species itself occurs naturally throughout much of eastern North America and is ecologically important partly because of calcium it delivers to food chains in deciduous forests (Baird, 1^2013^; Blair, 1982; Borer, Sapp, & Hutchinson, 2013; Holzmueller, Jose, Jenkins, Camp, & Long, 2006; Linzey & Brecht, 2003; Lovenshimer & Frick‐Ruppert, 2013). In addition, the plant is a cultural icon, serves as the emblem of three southern US states (Jordan, 2010), and is valued in the horticulture industry at 30 million dollars in annual sales (NASS USDA, 2007 Census of Agriculture). In the past three decades, *C. florida* populations have experienced major declines in health due to the introduction of a fungal pathogen (*Discula destructiva*) to North America (Miller, Masuya, Zhang, Walsh, & Zhang, 2016), the causal agent of dogwood anthracnose (Redlin, 1991). Northern and mountain populations have been hardest affected with up to 98% mortality occurring in monitored stands (Hiers & Evans, 1997; Jenkins & White, 2002; McEwan, Muller, Arthur, & Housman, 2000; Rossell, Rossell, Hining, & Anderson, 2001; Sherald, Stidham, Hadidian, & Hoeldtke, 1996; Williams & Moriarity, 1999). As dogwood anthracnose disease progresses southward along the Appalachian Mountains, populations of *C. florida* continue to decline (Jones, Smith, & Twardus, 2012). Whether or not the range of dogwood anthracnose (Figure 1) will expand to the southeast overtime is uncertain. Understanding the adaptive mechanisms in *C. florida* that may possibly limit the spread of disease will be important in the conservation of the species. Iridoid glycosides in particular are highly abundant in Cornelian taxa and have been noted to play roles in plant defense and disease resistance (Stermitz & Krull, 1998; Yue et al., 2006) in addition to various phenolic and tannin compounds (Dudt & Shure, 1993). In this work, we integrated evidence from multivariate analyses of flowering dogwood tree metabolomes, reduced genome sequences from genotype by sequencing (Pais, Whetten, & Xiang, 2016; Peterson, Weber, Kay, Fisher, & Hoekstra, 2012), and environmental data (model controls) to address the following questions: (1) What is the relationship between genetic diversity and chemodiversity? (2) Is there evidence from candidate SNPs and metabolites for local adaptation, and are there particular chemical biomarkers such as iridoid glycosides associated with either diseased or healthy plants? (3) Likewise, do healthier plants exhibit greater chemodiversity?

## 2 MATERIALS AND METHODS

### 2.1 Plant material

Natural populations of *C. florida* were sampled from the mountain, Piedmont, and Coastal Plain regions of North Carolina during the summer of 2012 (Pais et al., 2016). Each region contained two populations, and each population consisted of one or two collection sites, or subpopulations, of 30–15 individual trees, respectively, for a total of 180 mature and unique trees. Leaves were dried and stored in silica gel under standardized conditions. All samples were given an extended period to dry before extracting thermally stable metabolites from leaves.

### 2.2 Metabolite extraction

Metabolites were extracted following a modified protocol of Strauch, Svedin, Dilkes, Chapple, and Li (2015). For each tree, extraction material (20 mg) was selected from all sampled leaf tissue visibly free of mold or necrosis to minimize the chances of sampling metabolites that were unique to fungi, altered or degraded during collection. During the drying period, eight samples developed mold growth, which prohibited their use for metabolic study—leaving 172 remaining samples. Leaf tissue was ground with liquid nitrogen in a Retsch MM 400 oscillating mill for one min at 25 Hz. After grinding, two ml of 50% methanol was immediately applied to samples. Samples were then incubated in a water bath for 30 min at 60°C and allowed to cool one hour at 4°C to minimize potential precipitate (soluble in warm solution) from being transferred to final vials. After centrifuging for one min, remaining supernatant was transferred to a new vial via a filtered syringe tube.

### 2.3 Untargeted metabolite profiling

Untargeted metabolite profiling was performed on a G6530A Q‐TOF LC‐MS system (Agilent Technologies, Santa Clara, CA). Five microliters of leaf extract was injected onto an Agilent ZORBAX Eclipse Plus C18 column (3 × 100 mm, 1.8 μm). Metabolites were separated using a binary gradient of solvent A (0.1% formic acid in water) and solvent B (0.1% formic acid in acetonitrile) at a flow rate of 0.6/ml/min. The elution gradient started with a one‐min hold at 2% B, followed by ramping up to 45% B over 16 min, and then was increased to 90% B in one min and held at 90% B for 2.5 min. The acquisition of mass spectra was performed in negative mode for a m/z range from 100 to 1,600, with the following parameters: drying gas temperature, 300°C; drying gas flow rate, 7.0 L/min; nebulizer pressure, 40 psi; sheath gas temperature, 350°C; sheath gas flow rate, 10.0 L/min; Vcap, 3,500 V; nozzle voltage, 500 V; fragmentor, 150 V; skimmer, 65 V; OctopoleRFPeak, 750 V.

### 2.4 LC‐MS data processing

Raw data files obtained from LC‐MS experiments were converted to the mzData format using Agilent Masshunter software, grouped into directories by population, and then uploaded to the XCMS Online platform (Tautenhahn, Patti, Rinehart, & Siuzdak, 2012) for automatic metabolite detection and alignment. Metabolite features—peaks defined by mass‐to‐charge ratio (m/z), retention time (RT), and intensity—were extracted with optimized parameters: centWave method, minimum–maximum peak width = 8 and 30, signal‐to‐noise threshold = 30, mzdiff = 0.01, prefilter peaks = 3, prefilter intensity = 2,000, and noise filter = 0. As retention time variation between runs was minimal, the peaks were aligned across all the samples without RT correction, using the following parameters: bw = 5, mzwid = 0.025, and minfrac = 0.75. A list of 2,785 aligned peaks/features from 172 individuals was then exported from XCMS Online as a tab‐separated file. A preliminary PCA using autoscaled distances of individual peak areas and a distance to model (DModX) test (implemented in XCMS) detected individual UM19 as a significant outlier sample (possibly due to extraction error) to be removed. As one metabolite may give rise to multiple peaks including isotope, adduct, or fragment peaks, the 2,785 peaks were further grouped by peak intensity correlation and RT similarity using built‐in procedures from the XCMS pipeline (Tautenhahn et al., 2012). This resulted in 377 metabolites, which were represented by the largest peak within each group. Metabolite annotation was performed by searching the exact mass of detected metabolite features against the Knapsack and Metcyc databases (Caspi et al., 2014; Shinbo et al., 2006) using a ten ppm threshold.

### 2.5 Chemodiversity index calculation

Calculation of the richness diversity index (S; Whittaker, 1972) is primarily described in this study as its results have straightforward and biologically meaningful interpretations, which can be easily reconceptualized from the study of species diversity to the study of chemodiversity. For clarity, we first define richness in the context of a typical community ecology study before transferring the analogy to studying intraspecific chemodiversity. In a hypothetical field divided into multiple plots, species richness is the number of unique species in the field or each plot (Whittaker, 1972). Alpha (α) richness refers to the average richness of plots while the total number of unique species in the whole field is gamma (γ) richness. When appropriating these indices for studying chemodiversity, we treat each “species” as a metabolite and the “plot within a field” is represented by an individual plant's chromatogram, which represents the sum of all metabolite intensity peaks. More simply, richness is the metabolite count in an individual sample, and when richness is averaged among each tree in a subpopulation, subpopulations can be statistically compared by α‐richness (Whittaker, 1972). In contrast, γ‐richness represents total number of unique metabolites within a given subpopulation. As exploratory findings showed that γ‐richness was equal among subpopulations, we hereinafter refer to α‐richness when reporting chemical richness.

### 2.6 Genetic marker data

We used genotype by sequencing (GBS; Peterson et al., 2012) of two Illumina Hiseq libraries, de novo assembly into 90‐bp GBS tags with STACKS (Catchen, Amores, Hohenlohe, Cresko, & Postlethwait, 2011), latent factor mixed modeling [a genotype–environment association (GEA) method; Frichot, Schoville, Bouchard, & François, 2013], and two *F*_ST_ outlier methods (Excoffier, Hofer, & Foll, 2009; Foll & Gaggiotti, 2008) to classify putatively neutral SNPS and SNPs exhibiting varying support for being under selection (Pais et al., 2016). Putatively neutral reference SNPs were used to calculate marker‐based inbreeding coefficients (*F*; Keller, Visscher, & Goddard, 2011) and identity‐by‐state matrices using PLINK (Purcell et al., 2007). We added an inbreeding coefficient (*F*) variable into logistic models characterizing plant health and disease (see section 2.9) because we recognized the need to account for greater heterozygosity (fewer homozygous loci than expected) within an individual, which could affect plant health (Ouborg, Biere, & Mudde, 2000) by yielding more unique metabolites and raising plant potential to respond to novel pathogen effectors (screening hypothesis; Jones, Firn, & Malcolm, 1991). GBS samples were also reanalyzed with aid of a newly available *C. florida* draft genome (Dogwood Genome Project (NSF ID: 1444567), and the draft genome was used as an additional resource to predict candidate gene function by inspecting BLAST hit annotations surrounding SNPs of interest.

### 2.7 Environmental‐functional traits

We correlated climate–soil variables (obtained through collection site measurements and GIS extrapolation; Pais et al., 2016) and temperature‐precipitation estimates at time of collection (daily–monthly; PRISM Climate Group; extracted 30 January 2015) with chemical and genetic data (Table 1). Similarly, plant health scores were plotted against chemodiversity levels. Visual health‐diseased estimates were taken using the procedure of Mielke and Langdon (1986) based on the percent of tree canopy affected by leaf blotting, necrosis, or branch dieback. Additionally, five categorical scores obtained from this method were converted into a binary variable. Plants with scores of four and five were considered healthy while plants with scores of three and below were considered diseased. This recoded binary variable served as the response for mixed model logistic regressions. For further description how environmental variables were selected for multivariate modeling of chemodiversity levels, see Appendix S1 and additional justification of mixed logistic models as described further in methods.

**Table 1:** Chart of all predictors considered in current study accompanied by abbreviations used in main text

Variable	Abbreviation
Mean precipitation during month of collection	
Precipitation at day of collection	
Average temperature at month of collection	
Temperature at day of collection	Tcol
Health score (1–5)	No Abbreviation
Health score (binary)	No Abbreviation
Inbreeding coefficient	*F*
Osmometer reading	No Abbreviation
Average diameter by height	No Abbreviation
Canopy cover average	No Abbreviation
Proximity to water	No Abbreviation
Percent humic matter (soil)	HM
Weight–volume ratio (soil)	WV
Acidity (soil)	pH
Base saturation (soil)	BS
Exchangeable acidity (soil)	Ac
Cation exchange capacity	CEC
Phosphorus (soil)	P
Potassium (soil)	K
Calcium (soil)	Ca
Magnesium (soil)	Mg
Sulfur (soil)	S
Sodium (soil)	Na
Manganese (soil)	Mn
Copper (soil)	Cu
Zinc (soil)	Zn
Mean annual temperature	No Abbreviation
Mean monthly rainfall	No Abbreviation
Minimum temperature of January	Tmin1
Maximum temperature of July	Tmax7
Average monthly precipitation in June	Prec6
Average monthly precipitation in July	Prec7
Precipitation of driest month	Bio14
Frost‐free period	FFP
Length of growing period	LGP
Elevation	No Abbreviation
Longitude	No Abbreviation
Latitude	No Abbreviation

### 2.8 Characterizing general relationships of chemical structure and diversity to plant health and genetic diversity

We determined the general structure among sampled populations and the diversity of metabolites from multilocus genotype data. We first used discriminant analyses of principal components (DAPC) to identify collection sites that clustered together, according to SNP or metabolite abundance data. Using the R package adegenet (Jombart, 2008; Jombart, Devillard, & Balloux, 2010), we performed discriminant analysis (DA) on the optimal number of principal components (PC) to maximize among‐population variation and minimize within‐population variation. We estimated and analyzed PC scores separately from two different datasets (both scaled): reference SNPs aligning to the *C. florida* draft genome and abundance data (log‐transformed) for 377 metabolites. We conducted DAPC both by defining groups by collection sites and by allowing the program clustering algorithm to find optimal cluster number (K) without priors. Discriminant analyses of principal components does not require assumptions on a population genetic model (e.g., linkage equilibrium of markers) in contrast to programs like STRUCTURE (Pritchard, Stephens, & Donnelly, 2000) so DAPC has been widely adopted in recent population genetic studies (Buchalski et al., 2016; Cahill & Levinton, 2016; Grünwald & Goss, 2011). Its use for metabolic study is recent, but its efficacy in discriminating different biologically meaningful chemotype classes has been demonstrated and favored over other discriminant analysis methods under certain circumstances (Gromski et al., 2015; Scheitz et al., 2013).

Next, we estimated average genetic diversity and chemodiversity of each subpopulation. Rarefied‐allelic richness was calculated per site using the hierfstat R package (Goudet, 2005) and the same genetic dataset analyzed in DAPC. For each subpopulation, heterozygosity (expected and observed) and nucleotide diversity were recalculated from GBS markers in Pais et al. (2016) aligning to the newly developed draft genome of *C. florida*. Genetic diversity estimates from Pais et al. (2016) were recalculated as heterozygosity and nucleotide diversity were previously calculated independently among two different sequence libraries and having a draft genome eliminated complications of synthesizing two different de novo libraries. We note in results that new heterozygosity–nucleotide diversity estimates are congruent with previous findings in Pais et al. (2016). Chemodiversity indices derived from 377 metabolites were calculated per sample and averaged by subpopulation for correlation analyses, and subpopulations were compared with 95% confidence intervals for each genetic‐chemical diversity estimate.

We next tested for correlations between environmental gradients, genetic differentiation, and chemical distance using Mantel tests and linear regression. Full and partial Mantel tests (Legendre & Fortin, 1989) were implemented in the R package ecodist (Goslee & Urban, 2007) with 9,999 permutations and 500 bootstraps to determine the strength and significance of association between population‐level metabolic distance, genetic distance, and mean Euclidean distances of spatial and environmental variables [i.e., displacement of collection sites, precipitation of driest month (Bio14), and temperature at day of collection (Tcol); see Environmental‐functional traits continued in Appendix S1 for justification of environmental variables tested]. For these correlation analyses, we employed Arlequin (Excoffier & Lischer, 2010) and reference SNPs (putatively neutral SNPs from Pais et al., 2016) to create a matrix of linearized *F*_ST_ values between subpopulations. We then correlated this *F*_ST_ matrix to an analogous matrix describing population similarities and dissimilarities using metabolite data. To compare metabolic distances between subpopulations, we constructed an ANOSIM (Analysis of Similarities) R matrix from intersample Euclidean distances following the approach of Houshyani et al. (2012) and Kabouw, Biere, van der Putten, and van Dam (2009), 377 log‐transformed metabolites, and the program PAST (Hammer, Harper, & Ryan, 2001). An ANOSIM matrix is a reduced‐dimension matrix describing the similarity between pairs of subpopulations based on differences in abundances of multiple metabolites. Correlations of the *F*_ST_ matrix to the ANOSIM R matrix of population‐level metabolic distances were assessed using one‐tailed Mantel tests of Pearson's *r* coefficient.

The significance of simple linear regressions between individual‐specific chemodiversity levels, inbreeding coefficients, and all available environmental predictors was also assessed. In addition, we determined the best multivariate models describing general chemodiversity as the response (Appendix S1). For these initial regression models, we used chemodiversity indices based on all 2,785 chemical features as this allowed us to more reliably detect general differences in chemical richness among samples. However, we caution that inclusion of correlated chemical features may bias the calculation of chemodiversity indices, and as such, we delegate reporting and discussion of such results in Appendix S1.

### 2.9 Biomarker analyses

For identifying biomarkers associated with healthy versus diseased trees, we primarily used random forests (RF) tests and a logistic mixed model predicting disease states based on abundance data of each metabolite. Random forests tests were previously compared to partial least squares discriminant analysis (PLS‐DA), principal component discriminant analysis (DAPC), and support vector machines (SVM) for their ability to correctly assign samples to biologically based classes using metabolic data (Gromski et al., 2015). Logistic mixed models predicting healthy–diseased states of plants were also considered given the ability to statistically evaluate a Bonferroni correction and analytically control for inbreeding coefficient, temperature at collection, and random effects of collection site. More details on parameters for RF tests, justification of variable selection for logistic mixed modeling, and other biomarker tests compared in exploratory analyses (PLS‐DA, DAPC, and SVM) are available in Appendix S1 (Biomarker analyses continued).

### 2.10 Predicting metabolite–SNP networks

To understand patterns between chemical data and individual loci while controlling for sample structure and environmental variability, we employed a linear mixed model implemented in EMMAX (Kang et al., 2010). This model has been used in genomewide association (GWA) studies of *Arabidopsis thaliana* (Bac‐Molenaar, Fradin, Rienstra, Vreugdenhil, & Keurentjes, 2015; Fournier‐Level et al., 2011; Li, Huang, Bergelson, Nordborg, & Borevitz, 2010; Li et al., 2014; Strauch et al., 2015) because of its computational efficiency, and its ability to handle and control for population stratification (Price, Zaitlen, Reich, & Patterson, 2010) and environment. We tested for both SNP associations to each metabolite and for SNP associations to the property of chemical richness. We corrected for population structure by entering an identity‐by‐state matrix (created from neutral reference SNPs to describe pairwise relationships between individuals) into our model. For SNP association, we log‐transformed metabolite abundances prior to association study. For each corresponding GBS tag of a SNP, we noted any BLAST result, gene annotation (SWISS‐PROT, TAIR, or UNI‐PROT), and alignment match to the transcriptome (Zhang et al., 2013) or draft genome of *C. florida*.

Chemical–genotype associations were calculated in EMMAX with: (1) no covariates present; (2) the Bio14 covariate present; (3) the Tcol covariate present; or (4) both covariates present (see Environmental‐functional traits continued in Appendix S1 for justification of environmental controls specified). *p*‐Value distributions from output files were plotted using R package Haplin (Wilcox, Weinberg, & Lie, 1998) to assess Q–Q plots for each metabolite. Results were considered significant for genotype–metabolite associations passing a Bonferroni correction with an alpha value of 5%. Only results from normally distributed Q–Q plots were considered.

To explore the relationship between metabolites, we applied Gaussian graphical modeling (GGM) to the 377 metabolite dataset. Gaussian graphical modeling utilizes partial full‐order correlation coefficients to test for correlation between two metabolites while removing other metabolite effects. Justifications and additional considerations of GGM are further discussed in Appendix S1 (Gaussian graphical modeling continued).

### 2.11 Modeling health versus disease: Logistic mixed modeling with chemodiversity of a specific set of biomarkers

To assess whether chemodiversity of a putative terpenoid derivative network was related to the odds of being healthy versus diseased, we employed logistic mixed modeling (previously applied to single metabolites; see section 2.9) with chemical richness (or H′, D1, D2, E, and BP indices; see Chemodiversity indices continued in Appendix S1) specified as a fixed effect (recalculated from metabolites in the GGM‐associated group of iridoid glucosides; see Candidate metabolite–SNP network). In other words, we recalculated chemodiversity indices from a set of related iridoid derivatives and substituted the predictor representing a given metabolite abundance in our aforementioned mixed logistic model (see section 2.9) for a given chemodiversity index. Our justification for recalculating chemodiversity from metabolites in presumably related biological pathways was to compare the evenness or dominance in the accumulation of the specific set of metabolites between diseased and healthy plants. We also recalculated and examined chemodiversity among ten metabolites with common results among GWA and biomarker tests in exploratory analyses (Appendix S1; Table S5). Lastly, we tested interaction terms between chemodiversity and the other effects found to influence plant health (i.e., inbreeding coefficient, temperature at collection, and random effects of collection sites). Upon adding interaction terms for temperature at collection, we consistently found the interaction effects to be insignificant. The same applied when testing interactions to inbreeding coefficient. Thus, we removed interaction terms from our models.

## 3 RESULTS

### 3.1 Genetic markers

Of 1,631 GBS tags (containing 2,118 SNPs) consistently genotyped from two Hiseq libraries in Pais et al. (2016), we selected 1,860 SNPs for studying chemical–genotype associations. These SNPs passed a 5% minor allele frequency filter, a locus genotyping rate in 80% or greater of all samples, and Hardy–Weinberg exact tests implemented in Genepop (Rousset, 2008) indicating allele equilibrium in over half of the subpopulations. We selected one SNP per GBS tag to reduce linkage disequilibrium in our dataset, parsing final SNP number to 1,446. For GBS tags showing no evidence of being under selection from Pais et al. (2016), SNPs occurring closest to the *PstI* cut‐site were selected, leading to 1,171 SNPs as the neutral reference. For GBS tags showing any evidence of being under selection (Pais et al., 2016), SNPs with the highest estimated *F*_ST_ were included in the 1,446 SNP dataset for GWA to metabolites. Of those 1,446 SNPs, 1,163 SNPs occurred within GBS tags aligning to the *C. florida* draft genome and were applied to DAPC analyses.

### 3.2 General patterns of chemical‐genetic structure and diversity

**Figure 2:**
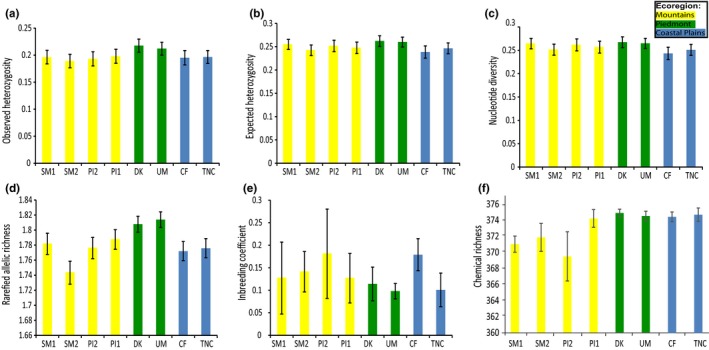
Comparison of subpopulation means of (a) observed and (b) expected heterozygosity, (c) nucleotide diversity, (d) rarefied‐allelic richness, (e) inbreeding coefficient, and (f) chemical richness calculated from 377 metabolites

**Figure 3:**
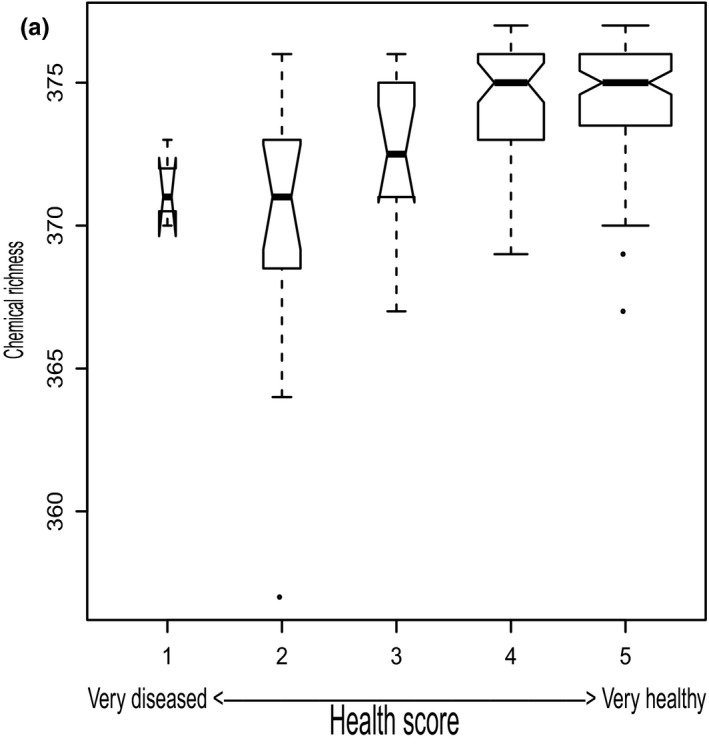
(a) Medians of individual‐level chemical richness values (calculated from 377 metabolites) by health score categories (coded 1‐5 with 5 being healthiest). Boxplots depict minimum and maximum values (whiskers), outliers (dots), first quartile, median, and third quartile. The notches in each box correspond to the 95% confidence interval of each median value, and the width of each box is proportional to the square root of each groups’ sample size, which represents three, 15, 14, 47, and 91 samples with health scores of one, two, three, four, and five, respectively

As shown in Pais et al. (2016) and corroborated by recalculations in this study, nucleotide diversity and heterozygosity levels were similar across sites (Figure 2a–c), but subsequent comparisons of rarefied‐allelic richness showed that Piedmont subpopulations had higher mean rarefied‐allelic richness than mountain and coastal subpopulations—especially in comparison with mountain subpopulation SM2, which had the lowest rarefied‐allelic richness (Figure 2d); contrasts of chemodiversity between subpopulations were most apparent for richness measures—with mountain subpopulations SM1 and SM2 having significantly lower chemical richness on average compared to Piedmont and coastal subpopulations (Figure 2f,g; seeFigure  S2 for subpopulation means of other chemodiversity indices). Levels of rarefied‐allelic richness and chemical richness were similar, and significant correlations between subpopulation means were observed (Figure S2). When grouping individual trees into healthy or diseased categories based on a one to five scoring system (Mielke & Langdon, 1986), the relationship of disease status to chemodiversity indices derived from 377 metabolites varied depending on the index (Appendix S1). In particular, chemical richness of the 377 metabolites highly overlapped among the five disease‐state groups (Figure 3), but it showed slight trends of medians increasing with increasing health states of individual trees (Figure 3).

**Figure 4:**
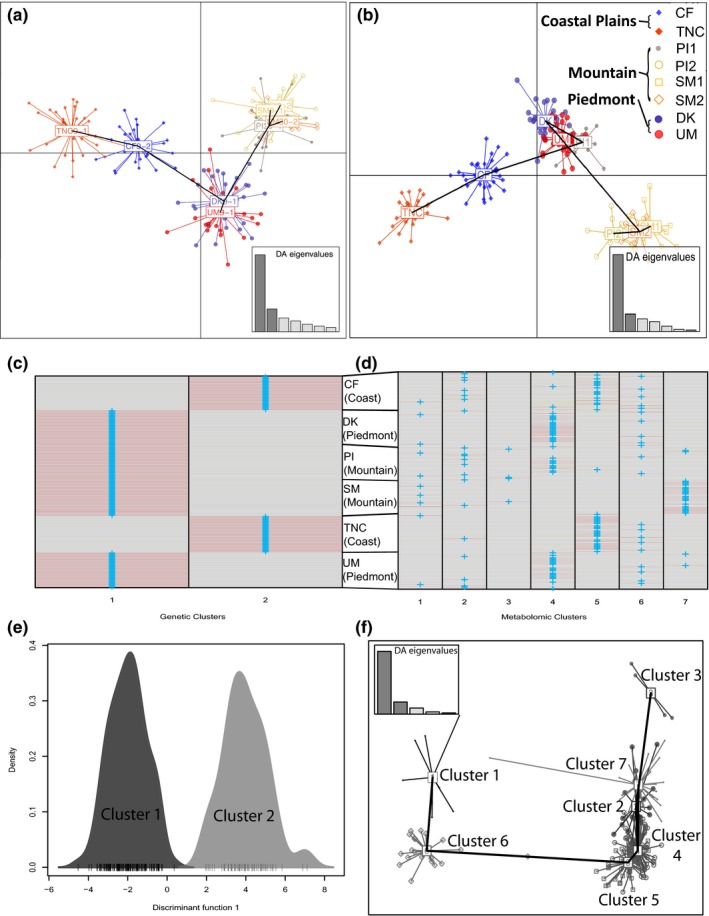
Plots of discriminant analyses of principal components (DAPC) derived from (a) 1,171 reference SNPs or (b) 377 chemical features (highest intensity metabolite per peak group). Dots of different colors and shapes represent individual trees belonging to different collection sites as indicated by legend. Mountain sites are as follows: Smoky Mountains (SM1 and SM2) and Pisgah Forest (PI1 and PI2). Piedmont sites are in Duke Forest (DK) and Umstead State Park (UM), and Coastal Plain sites are in the Croatan forest (CF) and the Nature Conservancy's Ecological Preserve at Nags Head Woods (TNC). When optimal cluster models are determined (i.e., when groups are not defined by collection site), assignment plots based on genetic distances (c) or metabolic distances (d) show cluster membership of each individual (rows with plus marks for most probable assignment). Optimal cluster models (determined by Bayesian criteria) are represented by scatterplots E and F for genetic and chemical distances, respectively

Subpopulation clustering patterns based on metabolic distances from discriminant analysis of principal components largely followed clustering patterns based on genetic distances (Figure 4). The only deviation was one mountain subpopulation from Pisgah forest (PI1) that clustered closer to Piedmont subpopulations, according to DAPC results of metabolic distances (Figure 4b). When the optimal clustering model was estimated without defining groups by subpopulations, DAPC using genetic distances only supported two clusters—one cluster consisting of samples from mountain–Piedmont ecoregions and another cluster consisting of samples from the Coastal Plains ecoregion (Pais et al., 2016; Figure 4c,e). In contrast, DAPC for metabolite data (without restricting samples to group by sampling location) supported up to seven clusters (Figure 4d,f) related to geography and environmental conditions at collection sites. The metabolic‐based clusters with high membership of mountain individuals (i.e., cluster seven and three) were located in the upper ordination space while cluster five (consisting of individuals primarily from the coast) and cluster four (including Piedmont samples and samples from the PI mountain subpopulation) were located lower along the ordination space (Figure 4f). Cluster one had relatively few coastal individuals; cluster six included no members of the SM mountain subpopulations; and cluster seven consisted primarily of SM mountain samples (Figure 4d). These results confirmed high sensitivity of metabolic data to environment (i.e., temperature), which was also supported by Mantel test results (Table 2) and regression model results of metabolic and environmental data (Tables S1 and S2, and Appendix S1). The remainder of reported results focus on metabolite associations with SNPs while controlling for the most important environmental factors influencing the general metabolic profile of samples as described in Appendix S1.

**Table 2:** Mantel tests of correlations between subpopulation‐level metabolic, genetic, and environmental distances. Chemical distance matrix obtained through Analysis of Similarities (ANOSIM) using 377 metabolites. Genetic distance matrix consists of linearized *F*_ST_ values calculated from 1,171 reference (putatively neutral) SNPs. Site‐level means of temperature at collection (Tcol) and precipitation of driest month (Bio14) used for matrices of Euclidean distances, and geographic distance among sites calculated from a *X*,* Y* coordinate system. Vertical bar denotes partial Mantel's test controlling for third matrix right of “|”

Mantel formula	Pearson *r*	*p* (*r* ≤ 0)
Metabolic distance versus geographic distance	.51134369	.02750275
Genetic distance versus geographic distance	.70352647	.00110011
Metabolic distance versus genetic distance	.3340171	.0670067
Metabolic distance versus Tcol	.64711201	.00050005
Metabolic distance versus Bio14	.41785384	.05830583
Metabolic distance versus genetic distance | geographic distance	−.04212434	.57645765
Metabolic distance versus genetic distance | Tcol	−.26949309	.88438844
Metabolic distance versus genetic distance | Bio14	.2555054	.1271127

### 3.3 Candidate metabolite–SNP associations

**Figure 5:**
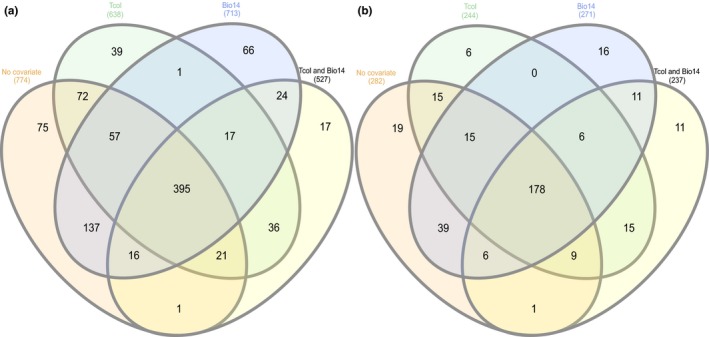
Total number of chemical features associated with SNPs. Venn diagram depicts overlapping results among various GWA tests with different covariates specified in the program EMMAX. Comparisons include overlapping results among (a) 975 significantly associated chemical features (of 2,785 chemical features) and (b) 346 significantly associated SNPs (of 1,446 SNPs). Covariates controlled for were: temperature at day of collection (Tcol) and precipitation of driest month (Bio14)

To understand the connections between genetic polymorphism and metabolite variation, we performed GWA analyses on all available SNPs and metabolites. As each SNP–metabolite association analysis was an independent test not biased by correlations among chemical features, each of the 2,785 chemical features of 377 metabolites was included. With and without the most important environmental covariates controlled for in GWA models, we identified 975 unique chemical features significantly associated with 347 unique SNPs. Overlapping chemical feature and SNP results among the various GWA tests (different covariates specified) are presented in Figure 5 and summarized here accordingly. The total number of chemical features and SNPs associated without covariates specified were 774 and 282, respectively. When Tcol (temperature at day of collection) was specified as a covariate, there were 638 chemical features significantly associated with 244 SNPs. When Bio14 (precipitation of driest month) was specified as a covariate, there were 713 chemical features significantly associated with 271 SNPs. Specifying both Tcol and Bio14 as covariates yielded 527 chemical features significantly associated with 237 SNPs. One SNP (B1567_16) was significantly associated with the property of chemical richness for all combinations covariate controls. Summary of similarities and differences in results among the various covariate‐dependent GWA tests for all chemical features and SNPs are available in Appendix S1.

**Figure 6:**
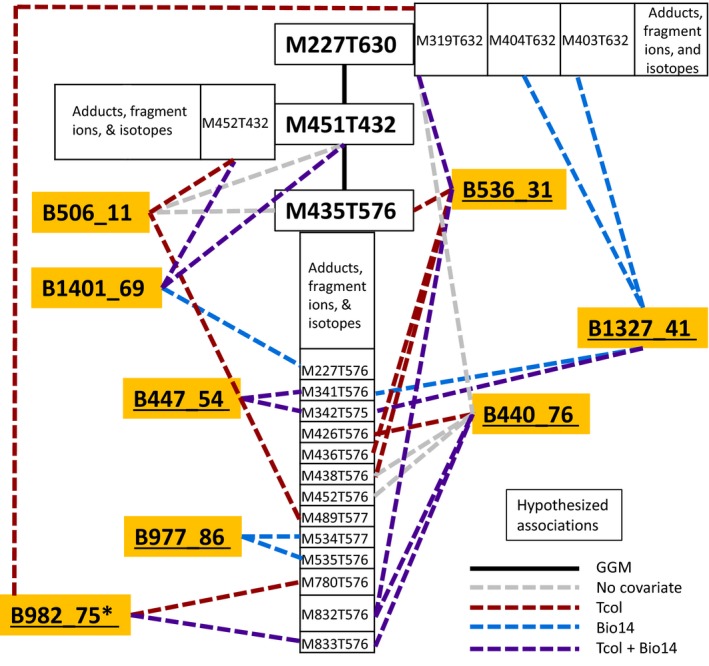
Summary of hypothesized SNP and iridoid glucoside associations of interest. (a) Predicted network connections of gene products and secondary metabolites were labeled based on genomewide association (GWA connections dashed) and Gaussian graphical modeling (GGM connections solid) results. SNPs are labeled starting with “B,” and chemical features are boxed and labeled by their mass‐to‐charge ratio (M) and retention time (T). Significant GWA connections with no covariate controlled for are highlighted gray. Significant GWA connections with at least temperature of collection (Tcol) controlled for are highlighted red, and significant GWA connections with precipitation mean of driest month (Bio14) controlled for are labeled blue. Significant GWA connections with both covariates controlled for are highlighted purple. SNPs associated with multiple chemical features are highlighted yellow and underlined, and a particularly noted SNP of interest (B982_75) is denoted with an asterisk

We performed GGM to formulate hypotheses of metabolite–metabolite associations based on partial correlation. This analysis, combined with the GWA results, revealed SNP–metabolite connections for a putative group of terpenoid glucosides (Figure 6a). We noted GGM connections of three metabolites consisting of an annotated iridoid glucoside/Eleganoside C (M435T576; Bailleul, Leveau, & Durand, 1981; Ali, Uzair, Krebs, Jahangir, & Habermehl, 2000; Xu, Wang, Zhang, & Yang, 2008), an aglycone/Cornolactone C (M227T630; He et al., 2014), and a possible intermediate (M451T432). Moreover, several SNPs were repeatedly found to be significantly associated with chemical features of these three metabolites (Figure 6a). SNP loci with multiple significant associations (labeled yellow; Figure 6) within this associated group were as follows: B506_11, B1401_69 (aligned to gene encoding for oligomeric Golgi complex; Ostertag, Stammler, Douchkov, Eichmann, & Hückelhoven, 2013), B447_54, B977_86, B982_75 (aligned to gene encoding for lectin‐domain receptor kinase; Singh & Zimmerli, 2013), B536_31, B1327_41, and B440_76 (aligned to gene encoding for a protein sensitive to rhizotoxicity; Sawaki et al., 2009; Fan, Lou, Yang, & Zheng, 2016). Several of these SNPs (B1401, B982_75, B440_76, B1401_69, B447_54, and B977_86) also showed evidence of being under selection in Pais et al. (2016).

### 3.4 Predicting disease status from metabolite markers

We identified 39 metabolites that were informative biomarkers for predicting plant disease status using RF (12), DAPC (8), PLS‐DA (12), SVM (11), or logistic mixed modeling (6) (Figures 8, S5, and S6). The majority of these biomarker metabolites accumulate more in healthy plants compared to diseased ones (Figures 8, S6, and S8). It is notable that there were few overlaps between the biomarkers detected by different methods (Figure S9a). Logistic mixed modeling of individual metabolites showed that six metabolites were significantly correlated with the log odds of being healthy versus diseased (Table 3) after controlling for temperature at collection, inbreeding coefficient, and collection site random effects. Of the 39 biomarker metabolites, five had significant associations with SNPs as revealed from GWA models controlling for environment (Figure S9a). The hypothesized SNP and GGM associations for these biomarkers are reported in Figure S10 along with results of greater focus concerning the hypothesized group of iridoid glycosides (Figure 6).

**Table 3:** Mixed logistic regression of individual metabolite effects (integrated intensity of chromatogram peak) on log odds of being healthy versus diseased. Model controls for inbreeding coefficient (*F*), temperature at collection (Tcol), and random effect of sites. Reporting six significant features of the 377 chemical features after retaining highest intensity metabolite per group of isotopic peaks

Chemical feature	Estimate	*SE*	*Z* value	*p* (>|*z*|)
M139T346	−1.65E‐05	5.89E‐06	−2.8	.005111
M277T1265	−1.33E‐04	6.75E‐05	−1.975	.04832
M301T1021	−4.60E‐05	2.28E‐05	−2.015	.04394
M307T406	−1.54E‐05	4.04E‐06	−3.825	.000131
M447T1161	3.92E‐05	1.84E‐05	2.131	.0331
M543T1327	−1.17E‐04	3.84E‐05	−3.038	.002378

**Figure 7:**
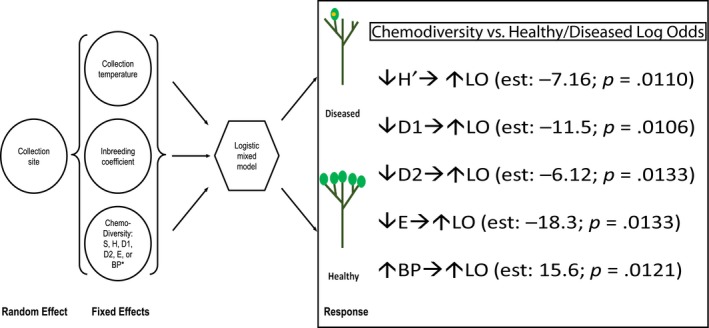
Depiction of chemodiversity trends [estimated from three unique metabolites in GGM‐associated group of iridoid glycosides: M227T630, M451T432, M435T576], representing six logistic mixed models controlling for inbreeding coefficient (*F*), temperature at collection (Tcol), and random site effects. Response is log odds (LO) of being healthy versus diseased

After controlling for inbreeding coefficient, collection site random effects, and temperature at collection, healthy‐diseased class correlations (log odds) to chemodiversity indices (calculated from the hypothesized GGM group of iridoid glycosides consisting of M227T630, M451T432, and M435T576) were significant for the majority of diversity indices (no differences in richness among samples and *p*‐values approximately .01 for H′, D1, D2, E, and BP indices). The Berger‐Parker (BP) dominance index (defined by the relative abundance of the most abundant metabolite per sample; see Chemodiversity indices continued in Appendix S1) was significantly correlated to the log odds of a plant being healthy versus diseased (Figure 7). The positive effect of the BP index was driven primarily by increasing abundances of M435T576, which was the most abundant metabolite within most samples (among other metabolites composing GGM network; Figure 6). Moreover, it reflected a greater unevenness of chemical expression for this metabolite in healthy plants relative to diseased plants (Figure 7). In other words, M435T576 was considered the most predictive biomarker among the two other associated iridoid glycosides (M227T630 and M451T432) for distinguishing plant health and disease.

Ten other credible biomarkers not correlated exclusively with the hypothesized group of iridoid glycosides were shown to be strongly associated with plant disease status (Figures S8 and S9). Accumulations of these ten metabolites (including M435T576) were highly predictive of plant health and disease status, according to multiple biomarker test results (Figure S9). Chemodiversity patterns derived from these ten biomarkers also showed that the dominance of M435T576 (in relation to the relatively even expression of other biomarkers) was associated with log odds of a plant being healthy versus diseased (Table S5; Appendix S1; Other candidate biomarkers continued).

## 4 DISCUSSION

### 4.1 Relationships of genetic diversity and chemodiversity

Our analysis showed largely concordant chemical‐genetic distance clustering patterns (Figure 4). For instance, mountain subpopulations (cooler in temperature and with higher disease severity) exhibited distinct chemical profiles and clustered together as in genetic analyses (excluding PI1), and two studied mountain subpopulations were lower in both metabolite richness (Figures 2f and S2a) and genetic diversity (rarefied‐allelic richness) than the studied Piedmont subpopulations (Figure 2d). Moreover, trees with greater individual‐level heterozygosity (i.e., smaller inbreeding coefficients) showed greater chemical α‐richness after controlling for other abiotic effects (Figure S7). These findings suggest that chemical variation preserved in dried leaves may have a link to genetic variation and functional diversity within a species.

There is clear impetus to conserve genetically and chemically diverse plant populations (Bustos‐Segura et al. 2017). It has been demonstrated that even small differences in genetic diversity (such as single amino acid substitutions) can yield large differences in chemical profiles (Kampranis et al., 2007), and several studies have found that higher intraspecific genetic diversity reduces herbivory and disease in plant populations (Hughes, Inouye, Johnson, Underwood, & Vellend, 2008). Moreover, emerging evidence has demonstrated how variation in intraspecific chemodiversity influences community diversity among different trophic interactions (Glassmire et al., 2016; Richards et al., 2015). In the context of this study, it is important to note the similarity between clustering patterns derived from genetic and metabolite data in addition to noting evidence that some diseased mountain subpopulations (Figure 1) have lower genetic and chemical diversity.

While dogwood disease may be constrained to the niche and life history of causal pathogens (Chellemi & Britton, 1992; Daughtrey, Hibben, Britton, Windham, & Redlin, 1996; Ennos, 2015; Holzmueller et al., 2006), *C. florida* does exhibit a gradient of variation in genetics and metabolites among North Carolina's coast, Piedmont, and mountain ecoregions (Figures 2, 4, and S1), which may correspond to variation in health scores (Figures S1 and S4; Pais et al., 2016). Furthermore, relatively high chemodiversity estimates among 377 metabolites are observed in healthier plants (Figure 3). As healthy plants occur in all environments from the Coastal Plains to mountains, this finding suggests that dogwood anthracnose may not necessarily be constrained by only abiotic factors (e.g., cooler and moister habitats) but instead may also be affected by both genetic and metabolite diversities. Genetic and metabolite diversity may be important to disease variation. High levels of metabolic and genetic diversity intrinsic to the host may benefit individual trees in staving off disease infection (Jones et al., 1991). Alternatively, low genetic diversity in mountain populations can also be a consequence of dogwood anthracnose disease effects (Hadziabdic et al., 2012). Although the co‐occurrence of these patterns in *C. florida* presents challenges to distinguish relative roles of abiotic, genetic, and chemical factors, available evidence supports an influence of genetics on disease as elaborated below.

**Figure 8:**
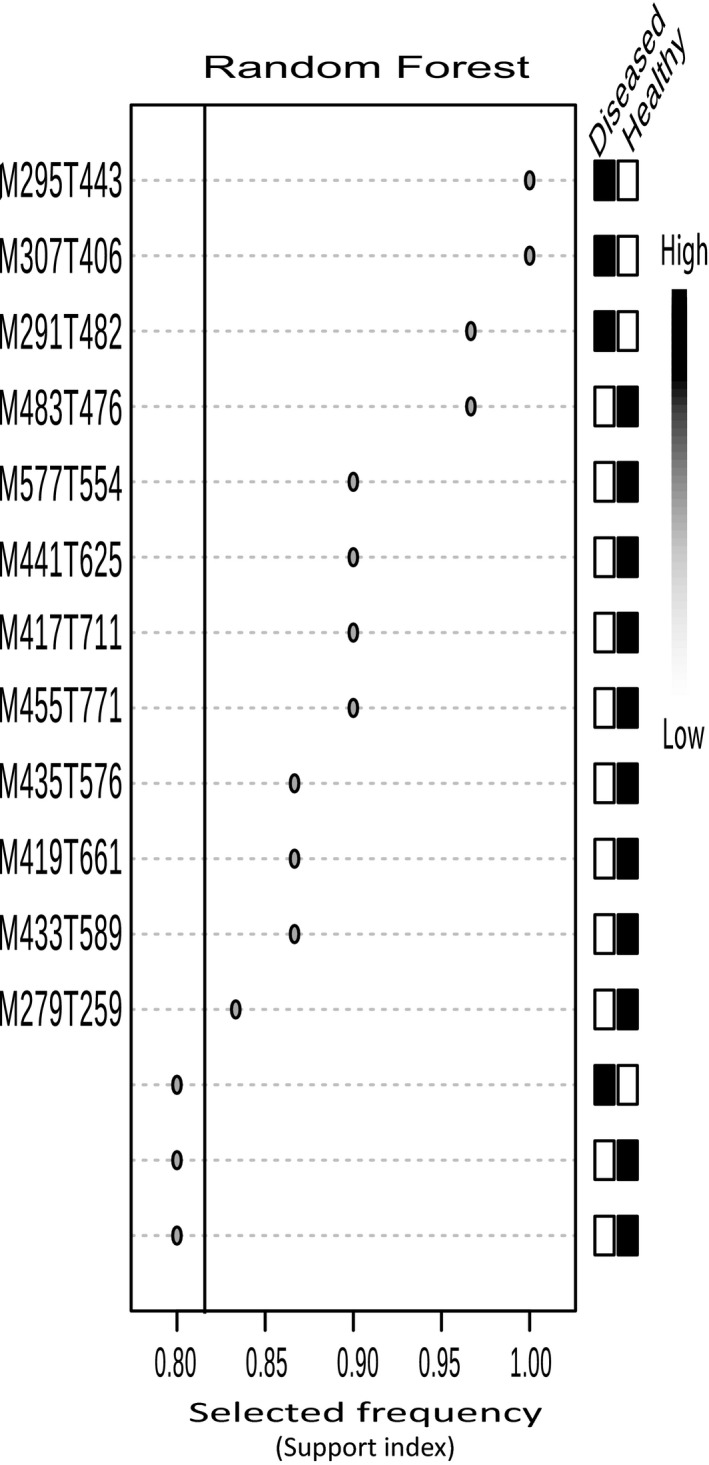
Top random forest (RF) results of biomarkers indicative of healthy or diseased plants. Metabolites arranged top to bottom based on ranked importance, and labeled metabolites right of solid black line on plot were considered for biomarker selection. Panel right of support index panel for biomarker tests indicates relative expression of each compound in healthy versus diseased plants with black shades representing higher expression and white shades representing lower expression

Previous ecological genomic analysis using GBS data (Pais et al., 2016) has identified SNPs under selection for local adaption in the species, and a few of these SNPs are associated with biomarker metabolites (predictors of plant health) after accounting for environmental covariates (Figure 6). In other words, our sampled subpopulations showed evidence of locally adapted genes associated with plant–chemical responses to disease pressure after controlling for environment. SNP loci B1401, B982_75, B440_76, B1401_69, B447_54, and B977_86 [previously identified to be under selection in Pais et al. (2016)] were found to be associated with a notable iridoid glucoside that was identified as a positive RF biomarker of health versus disease (M435T576; Figures 6, 8, S8, and S9). Several of these candidate SNPs occurred on loci with predicted functions related to disease resistance. Some SNP loci such as B1401 may encode for proteins (i.e., an oligomeric Golgi complex) that facilitate glycosylation to inhibit disease (Ostertag et al., 2013). Other loci such as *F*_ST_ outlier B982 (Pais et al., 2016) are predicted to encode for signaling receptors like lectin‐domain receptor kinases, which have been previously implicated in plant immunity responses among other signaling processes (Singh & Zimmerli, 2013), and recent functional experimentation on resistance genes encoding for such receptors in Solanaceous plants has provided evidence for resistance against *Phytophthora* disease (Wang, Weide, Govers, & Bouwmeester, 2015). Other SNP loci like B440 may encode regulatory proteins (Fan et al., 2016; Sawaki et al., 2009), which similarly respond to stress by regulating transcription of genes involved in pathways such as immunity response. Biomarker M435T576 and its related metabolites belong to a class of terpenoid derivatives that have known antimicrobial and antifungal properties (Bartsch et al., 2010; Chang, Xuan, Xu, & Zhang, 2001; Meng, Lu, Li, Yang, & Tan, 1999; Whitehead, Tiramani, & Bowers, 2016) and are likely sensitive to disease‐mediated signaling processes (Caplan, Padmanabhan, & Dinesh‐Kumar, 2008). For instance, M227T630 was identified as Cornolactone C, an iridoid isolated previously from *C. florida* (He et al., 2014), which belongs to a compound class known to accumulate in response to infection and has documented antimicrobial properties (Marak, Biere, & Van Damme, 2002).

These predicted chemical compounds serve as an important guide for prioritizing which SNPs and biomarkers to further test in future research. The abundance and inducibility of certain secondary metabolites such as flavonoids, other phenolics, and glycoside derivatives have been found to be heritable and mediated by herbivore–pathogen pressures (Johnson, Agrawal, Maron, & Salminen, 2009; Li et al., 2014). Determining the specific genetic factors regulating such adaptive metabolites remains an important goal, and this study adds to emerging efforts to integrate large secondary metabolite concentration data with information from genome scans (Eckert et al., 2012; Jensen, Foll, & Bernatchez, 2016; Talbot et al., 2016).

### 4.2 Herbivore/pathogen interactions influencing chemodiversity in plants

Explanations for natural variation of secondary metabolism have long been debated (Fraenkel, 1959). Intraspecific variation of plant chemodiversity can be attributed to differences in the environment (e.g., variation in surrounding plant, herbivore, fungal, or microbial communities as well as abiotic heterogeneity; Tahvanainen & Root, 1972; Root, 1973; Barbosa et al., 2009; Sardans et al., 2011; Rivas‐Ubach et al., 2014) or to genetic variation within the species. Proponents of the latter explanation cite observations used to argue for the role of chemodiversity in ecological function and heritability; namely, these authors note that congruent patterns of genetic diversity and chemodiversity are often inversely related to herbivory or infection levels (reviewed in Moore et al., 2014; Raguso et al., 2015). As an example, one study of *Smallanthus macroscyphus* (Asteraceae) reported less sesquiterpene lactone diversity (herbivory deterrent) in populations further away from the equator, which was explained as a result of selection for lower toxicity due to fewer herbivore–plant interactions in areas far from the equator (Aráoz, Mercado, Grau, & Catalán, 2016; Salazar & Marquis, 2012). This variation of sesquiterpene lactone diversity in the species may well have a genetic basis, which has not been investigated. In *C. florida*, a subset of the species distribution occurs from the southern Appalachians to southeastern Coastal Plains where an elevational‐temperature gradient spans eastward through the Piedmont, and we have found a decreasing trend of chemodiversity and genetic diversity in subpopulations more embedded in the Appalachian Mountains (Figure 2d,f,g). While lower temperatures may possibly be related to lower secondary plant metabolism of trees in the mountains, lower chemodiversity levels in mountain populations may be a result of lower genetic variation or a consequence of the disease infection, as mountain populations are in general less healthy. While diseased plants may show deficiencies in metabolism due to necrosis, our standardized method for extracting visibly unaffected tissue from both diseased and healthy plants makes the infection scenario less likely. The less healthy plants may possess genotypes that mediate metabolism of biosynthetic pathways in ways contributing to less constitutively expressed products—increasing plant susceptibility to initial infections. Higher genetic diversity in certain individuals (e.g., less homozygous genotypes; Figure S7) or subpopulations (e.g., higher rarefied‐allelic richness; Figure 2d) can confer a greater range of gene products (e.g., secondary compound precursors) and increase host ability to respond more readily to any general infection (Firn & Jones, 2000).

On the other hand, herbivory and infection on plants can also induce greater secondary compound diversity or induce dominance of certain compounds (Mithöfer & Boland, 2012; Thoss & Byers, 2006). While our study could not discriminate between constitutive and induced chemical diversity, our analysis of chemodiversity derived from a specific set of biomarkers did show that healthy plants tended to have greater unevenness of chemical expression than diseased plants (Figure 7). The unevenness seemed largely attributed to variation in expression of certain biomarkers (i.e., M435T576). In *C. florida*, candidate SNPs like B982_75 and B440_76 as well as biomarker M435T576 in the iridoid glucoside network (Figure 6) may represent examples of candidate genes governing variation in accumulation and degree of inducible expression for certain defense compounds.

## 5 CONCLUSIONS

Our study demonstrates untargeted metabolite profiling is a useful approach for understanding biodiversity in a new dimension. Secondary metabolites preserved in dried leaves of *C. florida* from natural populations provided data for evaluating chemodiversity and identifying potential disease biomarkers. We found congruent patterns of chemical and genetic variation and identified several biomarkers indicative of disease and health after accounting for the effects of environment. From those results, a select group of candidate SNPs and metabolites (i.e., iridoid glucosides) of clear ecological importance was identified to guide future study. Additional investigation of chemical diversity with increased sampling across the species range may provide more details on the relationship among genetics, metabolites, and dogwood anthracnose in *C. florida*, which in turn may shed light on forest diseases in general.

## CONFLICT OF INTEREST

None declared.

## AUTHOR CONTRIBUTIONS

With the support of Qiuyun Xiang and Xu Li, Andrew Pais conducted all collections, experiments, and analyses under the advice of the co‐authors and members of his dissertation committee. Xu Li supervised the metabolite profiling experiments and data analysis. This manuscript was drafted by Andrew as part of his PhD Dissertation and was edited by other co‐authors.

## DATA ACCESSIBILITY

Uploaded datasets for R analyses available on Dryad (https://doi.org/10.5061/dryad.15066), including (1) untransformed matrix of all 2,785 chemical features exported from XCMS; (2) matrices of all environmental‐functional‐genetic trait data used in multivariate models; and (3) Appendix S1 containing summary of all significant GWA results with a Bonferroni correction of 0.05 (Table S3) and list of compounds found in Knapsack and Metcyc databases that are similar in mass (delta ppm < 10) to notable chemical features reported in this manuscript (Table S4).
