# Supplementary material for: Discovering variation of secondary metabolite diversity and its relationship with disease resistance in Cornus florida L
Source: Ecol Evol. 2018 May 4;8(11):5619–36. doi: 10.1002/ece3.4090 (PMC6010843; doi:10.1002/ece3.4090)
Supplement: Supplementary file 1 [file ECE3-8-5619-s001.docx]

**Supporting Information**

*Chemodiversity equations*

Represented are six indices of chemodiversity described and applied from Morris *et al* (2014): Richness (S); Shannon’s diversity (H’), Simpson’s index of diversity (D1), Simpson’s inverse or dominance index (D2); evenness (E), and Berger-Parker’s index of dominance (BP). Formulas have been previously described in Shannon (1948), Simpson (1949), Hill (1973), and McCune and Grace (2002). Given that p_i_ is the relative proportion of total chromatogram intensity belonging to a chemical peak for a given sample, p_max_ is the proportion of total intensity attributable to the most abundant peak in a sample, and S is richness, formulas are defined as:

S = number of metabolite features (peaks) detected in a given sample.

_S_

H’ = $-\Sigma$(p_i_)(ln p_i_)

^i=1^

_S_

D1 = 1-$\Sigma$(p_i_)^2^

^i=1^

_S_

D2 = 1/$\Sigma$(p_i_)^2^

^i=1^

E = D2/S

BP = p_max_

*Chemodiversity indices continued*

We briefly describe the concept of richness in relation to other diversity indices from Hill (1973) and Morris *et al.* (2014). In the context of studying species diversity in a field subdivided into multiple plots, species with different relative abundances (p_i_) in each plot are resampled to estimate species diversity. Shannon’s index (Shannon 1948) takes into account both α-richness and evenness of p_i_ (degree of similarity-dissimilarity in p_i_ among species) within each plot compromising the field. The smallest Shannon’s index (H’) value is zero, meaning no other species are present or the evenness is so low that only one species is present. The maximum H’ value is the natural logarithm of species richness. Simpson’s index of diversity and Simpson’s inverse or dominance index also rise as species α-richness and plot evenness increase, albeit their minimum-maximum values and rates of change differ (McCune & Grace 2002). Simpson’s index of diversity represents the probability that resampling a plot yields different species—where zero represents a plot dominated by only one species and the maximum value (one) is reached when all possible species are present at equal proportions within a plot. Simpson’s inverse index—not to be confused with the reciprocal of Simpson’s index of diversity—has a minimum value of one (i.e. only one species present) while the maximum value is the total species number (when species’ relative abundances are equal). Lastly, Berger-Parker’s dominance index defines plot diversity by the most abundant species’ relative abundance (Berger & Parker 1970).

Appropriating diversity indices’ equations for quantifying chemodiversity is done when species’ relative abundances (p_i_) in a plot (comprising a field) are substituted for metabolites’ relative abundances (Randlkofer *et al.* 2010; Hilker 2014) within one individual (comprising part of a subpopulation). Shannon’s index applied to chemodiversity is consistent with usage of Shannon’s index in community ecology—representing uncertainty that an individual sampled from a plot (or standardized peak area sampled within an individual plant’s chromatogram) is the same species (or metabolite) when resampled from the plot (or chromatogram). Simpson’s index of chemodiversity is the probability any two random samplings of the same total chromatogram space represent different metabolites (McCune & Grace 2002). Simpson’s inverse or dominance index (Simpson 1949) is a similar metric of the Simpson’s index. To reiterate, an increase in metabolite richness (S) contributes to increases in Shannon’s index (H’), Simpson’s index (D1), and Simpson’s Inverse-Dominance index (D2), but a greater continuity among all metabolites’ expression levels within a sample also results in greater H’, D1, and D2 measures since these indices account for richness and relative abundance. Simpson’s evenness is calculated by dividing Simpson’s Inverse index (D2) by chemical richness (D2/S; Smith & Wilson 1996) to measure evenness of metabolites’ expressions. The Berger-Parker (BP) index we applied to our chemical data represents the most abundant metabolite’s relative abundance in relation to other metabolites in a sample (Berger & Parker 1970), and it is inversely related to evenness.

There were minor differences among subpopulation means regarding various evenness-based indices of chemodiversity (Fig. S2), and trends of rising health scores with chemodiversity were observed for H, D1, D2, and E indices. However, chemodiversity was maximum at health scores of three for these indices (Fig. S3). When comparing BP (the abundance of the metabolite with the greatest relative abundance among the 2,785 chemical features per individual) to disease status, a trend of decreasing dominance (reflected by decreasing BP) and increasing health scores was observed—reflecting the most abundant metabolite’s higher dominance being associated with less chemodiversity and greater susceptibility of plants to disease (Fig. S3). While trees with the lowest health score of one had significantly higher BP (one metabolite’s dominance) than trees with higher health scores, it was unclear if these relationships were significant among trees with health states of two, three, four, and five (Fig. S3).

*Environmental-functional traits continued*

Mantel test results showed strong collinearity between site-specific effects on chemical differences between subpopulations (Table 2). Isolation by distance tests using either genetic distances or metabolic distances for Mantel tests were both significant (r > 0.5 and p < 0.05). The relationship between metabolic distance and genetic distances of subpopulations was not significant (p = 0.0670), and it was clearly insignificant when incorporating geographic distance, temperature at collection, or precipitation of driest month as controls (Table 2). A mantel test comparing metabolic distances and mean differences in temperature at collection was the most significant with a Pearson r coefficient of 0.647 and a p-value of 0.0005.

When analyzing environmental predictors in simple linear regression, several predictors had significant correlations to at least three or more chemodiversity (Fig. S4). In this select instance, all chemical features were considered to characterize the influence of each environmental predictor on the general metabolic profile of each sample. Such predictors were considered in variable selection for best multivariate models describing general chemodiversity. Our best multivariate model indicated that higher inbreeding coefficients in individuals were predictive of smaller chemical richness after controlling for other abiotic effects (Estimate = -47.276 chemical features, p = 0.007; Fig. S7).

Variables were chosen selectively for multivariate models based on three criteria: (1) Reduction of collinearity so that variance inflation factor was less than 10 for remaining variables; (2) calculation of AIC scores for competing models with R package glmulti (Calcagno 2013); (3) and relevance of predictors for explaining our hypotheses of interest. For instance, in our analyses of genome-wide association using the program EMMAX (Kang *et al.* 2010), we incorporated the mean annual precipitation of the driest month (Bio14), mean temperature during day of tissue collection (Tcol), or both into our various mixed models testing for associations of SNPs to specific chemical phenotypes. The reasons for choosing these two variables for this particular GWA model (see *Predicting metabolite-SNP networks* for more details) were as follows: (1) These two environmental attributes had no missing values for 165 of 171 individuals; (2) after reducing collinearity between other environmental attributes to a variance inflation factor score of 10, TCol had the strongest joint-biplot correlation out of the remaining variables to a non-metric dimensional scaling (NMDS) plot of chemical phenotypes (Fig. S1); (3) Bio14 was chosen as another predictor because the direction of its joint-biplot correlation to the NMDS plot was markedly different from Tcol and its covariates (Fig. S1).

*General patterns of chemical-genetic structure and diversity continued*

For multiple linear regression, we used the R package glmulti (Calcagno 2013) to select the best model (based on AIC score and a genetic algorithm of 500 for population size) from the set of predictors reduced of collinearity (VIF<10) that were also significantly correlated to at least three of six chemodiversity indices according to simple linear regression. The multivariate model with the best AIC score to predict variables’ effect on richness of 2,785 chemical features included individual-level inbreeding coefficient, a covariate of precipitation (mean precipitation during month of collection), a temperature covariate (FFP), and a random effect by collection site. All three main effects were found to be significant, but the effect size of inbreeding coefficient on chemical richness was greater compared to the other two main effects controlled for in the model (Estimate = -47.276, p = 0.007). Predictions of inbreeding coefficients’ effect on general chemical richness (with other variables held at median values) were plotted in Fig. S7. While less informative to the questions posed in the introduction, reports on the best multivariate linear models corresponding to other chemodiversity indices were reported in Table S2.

*Biomarker analyses continued*

For biomarker tests identifying features indicative of health or disease, we provide further details of parameterization. Using the 377 representative chemical phenotypes without adducts, fragment ions, isotopic peaks, DAPC was run similarly as when applied to analyses of general population structure, and PLS-DA, SVM, and RF were implemented on the same dataset using the Metaboanalyst online platform (Xia *et al.* 2015). Significant correlations from the logistic mixed model in addition to significant results from DAPC, PLS-DA, SVM, and RF were tallied and cross-referenced with SNP-metabolite associations to identify notable metabolites. DAPC was implemented in an R environment while the other biomarker tests were implemented via an online web tool available at www.metaboanalyst.ca.

For DAPC using 377 metabolomic features (log-transformed), we determined how defined-subpopulations related to one another, how many clusters existed without defining groups, and searched for biomarkers that adequately discriminated between healthy and diseased plants. For visualizing scatter plots of how defined populations were related to one another, we utilized the first 55 principal components [optimal according to root mean squared error comparisons (RMSE)] and seven discriminant functions, but for determining the number of optimal clusters for DAPC without group priors, we utilized the maximum number of principal components. Most importantly, we used the first 45 principal components that were optimal (according to RMSE) for discriminating between healthy and disease groups of samples. With DAPC of health vs. disease, we examined the loading plot of chemical features contributing most to the discrimination of groups. A two percent contribution threshold was considered sufficient for identifying biomarkers of health vs. disease in DAPC since such a threshold corresponded approximately to three standard deviations above the average contribution value (99.7 percentile group). Since we were selecting biomarkers for further consideration based on overlapping findings from other biomarker tests, this z-score distribution threshold was more relaxed than a z-score corresponding to a typical Bonferroni correction.

For PLS-DA, SVM, and RF tests, the table of 377 peak intensities was log-transformed using a generalized logarithm transformation provided in the MetaboAnalyst web platform. The optimal number of components to use for PLS-DA was found to be four according to scores of accuracy and Q2 for a ten-fold cross-validation method. For determining important biomarkers indicated by PLS-DA, we ranked features by the weighted sum of absolute regression coefficients and selected the first dozen candidates before changes in coefficient values began to plateau. The SVM algorithm was used to classify samples into disease vs. healthy groups using the greatest number of variables achieving the highest overall predictive accuracy and a built-in ranking method for features. The top eleven features were chosen as biomarkers as these features predicted the correct class most frequently. Beyond the first eleven biomarkers, changes in selected frequency were negligible. We also classified groups with RF, chose the model with most features yielding highest overall predictive accuracy, and used a built-in ranking method based on selected frequency to select the top dozen chemical features for consideration as biomarkers.

The logistic mixed model controlling for inbreeding coefficient, temperature at collection, and a random site effect was parameterized and executed with functions from the R package ordinal. We designed our model to find individual metabolites that were associated with higher log odds of being healthy vs. diseased after controlling for fixed and random effects. Since temperature at collection was found to influence chemical expression the most (Fig. S1, Fig. S4, Table S1), we also parameterized that variable as a fixed effect to control for while treating each population as a random effect to capture other environmental variables unique to the location and time of collection.

*Gaussian graphical modelling continued*

GGM correlations produce hypotheses of metabolite networks based on chemical expression differences intrinsic to the dataset. This *de novo* method for reconstructing regulatory networks (Krumsiek *et al.* 2011) has been applied the study of metabolite networks in tomatoes (Bénard *et al.* 2015) and has been expanded to associate chemical expression to specific genotypes (Krumsiek *et al.* 2012). Considerations of GGM and its variants are reviewed in Bartel *et al.* 2013, and we note that the approach we have adopted is appropriate to this study given our sampling effort and implementation of the model.

Unlike prior analyses where our matrices of chemical phenotypes had true values of zero, the constraints of GGM required us to impute missing data using a random forest algorithm (Stekhoven 2012). To overcome the statistical requirement for immense sample sizes to reconstruct metabolomic networks, we employed a shrinkage estimation approach implemented in the GeneNet R package (Schäfer and Strimmer 2005) to allow for the calculation and reliable interpretation of GGMs from our matrix. After calculating partial correlation coefficients, significant correlations that passed a Bonferroni correction with a cutoff of α = 0.05 were retained and plotted with our hypothesized SNP-metabolite networks.

*Other candidate biomarkers continued*

For our set of ten metabolites with significant GWA results and considered biomarkers indicative of health vs. disease, we found that H’, D1, D2, E, and BP were significantly associated with the log odds of a plant being healthy vs. diseased after controlling for inbreeding coefficient, random effects of collection site, and temperature at collection (latter being consistently significant). While all ten biomarkers together were predictive of diseased vs. healthy states (Fig. S9C), as illustrated in Fig. S9B and Fig. S8, some biomarkers were more associated with health or disease than other phenotypes in the set of ten. Therefore, the negative effect H’, D1, D2, and E increases had on the log odds of plant healthy (Table S5) was expected to reflect the relative unevenness of metabolite accumulation in healthy plants compared to diseased plants. In other words, diseased plants had a closer to even expression profile of these ten biomarkers than healthy plants. On other hand, the rise in BP (Table S5) had a positive effect on the log odds of being healthy. The rise in this index was related to the rise in abundance of the phenotype with the highest relative abundance of the ten other biomarkers. This phenotype (M435T576) was also the most abundant phenotype in the set of three biomarkers constituting the hypothesized glucoside network.

**Supplementary Figures and Tables**

Figure S1. Scatterplot of tree samples and their metabolomics distances from each other based on nonmetric dimension scaling (NMDS) of 285 log-transformed chemical features. Criteria for selecting samples and features for NMDS: retaining only highest intensity metabolite of isotopic peak group, retaining metabolites with at least one pairwise difference between collection sites, and filtering out samples or metabolites with high abundance of zero-values. Black lines are joint bi-plots, which represent abiotic elements most correlated with different planes of ordination to chemical features. Samples colored by collection site, and labelled samples represent diseased individuals.

Figure S2. Comparison of subpopulation means of chemodiversity indices calculated from 2785 chemical features, including (A) chemical richness, (B) Shannon’s index, (C) Simpson’s index of diversity, (D) Simpson’s inverse index, (E) evenness, and (F) Berger-Parker’s index. Black whiskers represent 95% confidence intervals of mean. Also plotted is a (G) simple linear regression between rarefied-allelic richness means and chemical richness means (of 2,785 chemical features) from eight subpopulations—spanning mountain (yellow), Piedmont (green), and Coastal Plain (blue) ecoregions of North Carolina.

Figure S3. Medians of chemodiversity indices by health scores (coded 1-5 with 5 being healthiest) and histograms of chemodiversity indices for datasets derived from all 2785 chemical features. Chemodiversity indices represented here are Shannon diversity (H’), Simpson’s diversity (D1), Simpson’s dominance (D2), evenness (E), and Berger-Parker dominance (BP). Boxplots depict minimum and maximum values (whiskers), outliers (dots), first quartile, median, and third quartile. The notches in each box correspond to the 95% confidence interval of each median value, and the width of each box is proportional to the square root of each groups’ sample size.

Figure S4. Scatter plots of eleven environmental-functional traits against six indices of chemical diversity: richness (S), Shannon diversity (H’), Simpson’s index of diversity (D1), Simpson’s dominance (D2), evenness (E), and Berger-Parker dominance (BP). Indices derived from all 2785 features. Red loess lines fitted for visualization of trends. Relationships boxed in red are significant according to simple linear regression, and only predictors with at least three out of six significant correlations to the diversity indices are represented. Tables of simple linear regression results for all predictors reported in Table S1. Abbreviations are: *F* (inbreeding coefficient), tcol (temperature at collection), ppt of month (precipitation at month of collection), dbh (diameter-by-height), and FFP (frost free period).

Figure S5. Density plot of samples representing discriminant analysis of principal components (DAPC) results, based on data from 377 metabolites with groups defined as samples that are healthy (green) or diseased (red). (B) Loading plot showing metabolites contributing most for distinguishing healthy vs. diseased plants.

Figure S6. Top results of biomarkers indicative of healthy or diseased plants from (A) Partial least squares discriminant analysis (PLS-DA), and (B) support vector machines (SVM). Metabolites arranged top to bottom based on ranked importance, and labelled metabolites right of solid black line on each plot were considered for biomarker selection. Panel right of support index panel for biomarker tests indicates relative expression of each compound in healthy vs. diseased plants with black shades representing higher expression and white shades representing lower expression.

Figure S7. Prediction plot of multiple linear regression with richness (S) of 2785 chemical features plotted as response and inbreeding coefficient plotted along x-axis. Both within subpopulations and among all samples, increases in inbreeding coefficients are related to decreases in chemical richness. Fixed effects of best model (determined by model selection with AIC criteria) are inbreeding coefficient, mean precipitation during month of collection, and frost free period. Site of collection treated as random effect and represented by each black line with total prediction line of all samples depicted as wider line. Green points represent plants visually categorized to be healthy while red points represent diseased samples. Predictions of mixed model generated in R environment by holding predictors constant at median while spanning minimum and maximum values of inbreeding coefficient.

Figure S8. Receiver operating characteristic (ROC) curves and histograms of ten selected log-transformed chemical biomarkers. These biomarkers were used to estimate interactions of chemodiversity and inbreeding coefficients and their effect on plant health.

Figure S9. (A) Venn diagram summarizing other notable biomarker results from DAPC, PLS-DA, SVM, RF, GWAS, and mixed logistic regression of individual metabolites. Biomarkers detected by at least two different tests highlighted in black boxes, and those ten consistently identified biomarkers are featured in (B) boxplot of chemical abundances. (C) Area under receiver operating characteristic curve (AUROC) derived from the ten selected biomarkers of disease-health. Blue shaded region is 95 percent confidence interval. Grey lines represent 1000 permutations using random data to assess true departure from null distribution (p < 0.001). Confusion matrix depicts credibility rate for correctly predicting individuals as either healthy or diseased. Credibility rate is (24 + 122)/170.

Figure S10. Other notable metabolites (M and T denoting mass-to-charge ration and retention time) significantly associated to SNPs (labelled starting with “B”) from GWA tests and passing at least one biomarker test predictive of health vs. disease. To simplify presentation, no adducts, fragments, or isotopic peaks shown here.

Table S1. Simple linear regression results [estimate,std error, F, P, r2] of all predictors. Response variables include chemodiversity indices estimated from all 2785 features including: richness (S), Shannon's diversity (H'), Simpson's diversity (D1), Simpson's dominance (D2), Simpson's evenness (E), and Berger-Parker's dominance (BP). Significant P values bolded along with r2 values above 0.1. First part of spreadsheet reports notable predictors with at least three out of six associations to chemodiversity indices shown. Second portion of spreadsheet contains reports on additional predictors in study.

Table S2. Multiple linear regression with chemodiversity indices (derived from 2785 metabolites) designated as response. Fixed effects of best model determined for each model of the six response variables of chemodiversity. Model selection based on AIC score. Random effect of sites incorporated afterwards into each of the best models.

Table S3. Summary of all significant GWA results with a Bonferroni correction of 0.05. Metabolite identifiers listed according to mass-to-charge (M) and retention time (T) properties. SNP identifiers start with label “B”, which is consistent with annotation from Pais *et al.* 2016. Results include tests do with no covariates implemented or include tests with the following covariates controlled for: Temperature at collection (tcol), precipitation mean of driest month (Bio14), or both covariates.

Table S4. List of compounds found in Knapsack and Metcyc databases that are similar in mass (delta ppm < 10) to notable chemical features reported in this manuscript.

Table S5. Logistic mixed models of chemodiversity indices estimated from ten features consistently found to be biomarkers associated to SNPs or health vs. disease. Table depicts the main effects of inbreeding coefficient (*F*), temperature at collection (tcol), and each of the six chemodiversity indices on log odds of being healthy vs. diseased after controlling for random effect of sites.
